# Draft Genome Sequence of an Extensively Drug-Resistant *Mycobacterium tuberculosis* Clinical Isolate of the Ural Strain OSDD493

**DOI:** 10.1128/genomeA.00928-13

**Published:** 2013-11-07

**Authors:** Shamsudheen Karuthedath Vellarikkal, Ajay Vir Singh, Pravin Kumar Singh, Parul Garg, Viswa Mohan Katoch, Kiran Katoch, Open Source Drug Discovery Consortium, D. S. Chauhan, Vinod Scaria, Sridhar Sivasubbu

**Affiliations:** Genomics and Molecular Medicine, CSIR Institute of Genomics and Integrative Biology, Delhi, Indiaa; GN Ramachandran Knowledge Center for Genome Informatics, CSIR Institute of Genomics and Integrative Biology, Delhi, Indiab; National JALMA Institute of Leprosy and Other Mycobacterial Diseases, Tajganj, Agra, Indiac; CSIR Open Source Drug Discovery Unit, Anusandhan Bhavan, New Delhi, Indiad

## Abstract

We describe the genome sequencing and analysis of a clinical isolate of *Mycobacterium tuberculosis* belonging to the Ural strain OSDD493 from India.

## GENOME ANNOUNCEMENT

Tuberculosis is caused by a closely related group of pathogenic organisms known as the *Mycobacterium tuberculosis* complex. Distinct lineages of *Mycobacterium tuberculosis* have been reported and have been characterized and classified based on spoligotype patterns. The six major distinct lineages are the Indo-Oceanic (EAI), East Asian (Beijing), East African-Indian (CAS), Euro-American (Haarlem, LAM, T, X), West African I (AFRI1), and West African lineage II (AFRI2) lineages (1). The Ural spoligotype was initially identified from the Ural region in Russia, which has one of the highest incidences of tuberculosis in the country. Nevertheless, the Ural spoligotype forms a minority of genotypes of *M. tuberculosis* strains isolated from this region (2). The genotype has also been reported from across Eurasia and Central Asia and forms a significant proportion of genotypes observed in some of these regions. The Ural genotype is thought to be associated with significantly low transmissibility, pathogenicity, and frequency of drug resistance (3, 4).

Understanding the genome sequence of the Ural strain of *Mycobacterium tuberculosis* would provide immense insights into the genomic architecture associated with low pathogenicity. In this paper, we describe the draft genome sequence of an extensively drug-resistant clinical isolate of *Mycobacterium tuberculosis* conforming to a novel spoligotype clustering within the Ural spoligotype. The clinical isolate OSDD493 was obtained from the strain repository maintained at the National JALMA Institute of Leprosy and other Mycobacterial Diseases, which is part of the Open Source Drug Discovery Open Access Repository. Spoligotyping was performed and drug sensitivity was evaluated per standard protocols (5–7). Drug sensitivity analysis revealed the isolate to be resistant to streptomycin, rifampin, isoniazid, ethambutol, ofloxacin, kanamycin, and ethionamide and sensitive to amikacin, pyrazinamide, capreomycin, cycloserine, and *para*-aminosalicylate sodium (PAS). DNA was isolated per standard protocols. The raw sequence data were generated after library preparation on Ion Torrent PGM according to protocols recommended by the manufacturers. Draft genomes were assembled de novo using CLC Genomics Workbench 6. The assembly resulted in 193 contigs at *N*_50_ values of 42,650 bp and a total assembly of 4,227,747 bp. Further, automated gene prediction on the draft genomes was performed using the RAST server (8). Analysis revealed 4,223 genes, including 42 RNA genes.

### Nucleotide sequence accession numbers.

This whole-genome shotgun project has been deposited at DDBJ/EMBL/GenBank under the accession number AVQJ00000000. The version described in this paper is version AVQJ01000000.
